# Pathogenesis of Lafora Disease: Transition of Soluble Glycogen to Insoluble Polyglucosan

**DOI:** 10.3390/ijms18081743

**Published:** 2017-08-11

**Authors:** Mitchell A. Sullivan, Silvia Nitschke, Martin Steup, Berge A. Minassian, Felix Nitschke

**Affiliations:** 1 Program in Genetics and Genome Biology, The Hospital for Sick Children Research Institute, 686 Bay Street, Toronto, ON M5G 0A4, Canada; mitchsullo@gmail.com (M.A.S.); silvia.nitschke@sickkids.ca (S.N.); msteup@uni-potsdam.de (M.S.); Berge.Minassian@UTSouthwestern.edu (B.A.M.); 2 Glycation and Diabetes, Mater Research Institute, Translational Research Institute, The University of Queensland, 37 Kent St., Woolloongabba, Brisbane 4102, Australia; 3 The Impact Centre of the University of Toronto, 60 St. George Street, Toronto, ON M5S 1A7, Canada; 4 Faculty of Mathematics and Science, University of Potsdam, 14476 Potsdam-Golm, Germany; 5 Division of Neurology, Department of Pediatrics, University of Texas Southwestern, 5323 Harry Hines Blvd., Dallas, TX 75390-9063, USA

**Keywords:** lafora disease, laforin, malin, polyglucosan body, chain length distribution, glycogen phosphorylation

## Abstract

Lafora disease (LD, OMIM #254780) is a rare, recessively inherited neurodegenerative disease with adolescent onset, resulting in progressive myoclonus epilepsy which is fatal usually within ten years of symptom onset. The disease is caused by loss-of-function mutations in either of the two genes *EPM2A* (laforin) or *EPM2B* (malin). It characteristically involves the accumulation of insoluble glycogen-derived particles, named Lafora bodies (LBs), which are considered neurotoxic and causative of the disease. The pathogenesis of LD is therefore centred on the question of how insoluble LBs emerge from soluble glycogen. Recent data clearly show that an abnormal glycogen chain length distribution, but neither hyperphosphorylation nor impairment of general autophagy, strictly correlates with glycogen accumulation and the presence of LBs. This review summarizes results obtained with patients, mouse models, and cell lines and consolidates apparent paradoxes in the LD literature. Based on the growing body of evidence, it proposes that LD is predominantly caused by an impairment in chain-length regulation affecting only a small proportion of the cellular glycogen. A better grasp of LD pathogenesis will further develop our understanding of glycogen metabolism and structure. It will also facilitate the development of clinical interventions that appropriately target the underlying cause of LD.

## 1. Introduction

Lafora disease (LD) is an autosomal recessively inherited disease that results from mutations in either the gene encoding for laforin (*EPM2A*) [1,2] or malin (*EPM2B*, also called *NHLRC1*) [3]. By 2009, over 200 independent families were found to suffer from LD with almost 100 distinct mutations in the two genes [4]. Since then, more mutations have been discovered [5,6]. While these two genes account for the majority of LD cases, there is evidence for at least one additional locus that may account for the remaining cases where neither *EPM2A* nor *EPM2B* are mutated [7].

The clinical outcomes of loss-of-function mutations in either *EPM2A* or *EPM2B* are generally similar, suggesting a closely related role of the two proteins laforin and malin in preventing LD. First epileptic episodes usually occur in adolescence. Subsequently, the disease progresses with ever worsening, increasingly intractable myoclonic seizures and cognitive decline through neurodegeneration. Eventually patients are bedridden, enduring in a vegetative state and finally succumbing to the disease, usually within ten years of symptom onset. Some studies have reported deviations from this general disease progression in patients with mutations in *EPM2A* or *EPM2B*, describing patients with a later onset or different rate of progression, e.g., a slower progression in particular patients with *EPM2B* mutations [8,9,10,11].

A hallmark of LD is the accumulation of Lafora bodies (LBs) in various tissues, including skin, skeletal muscle, heart, liver, and brain. LBs are stained on tissue sections with Schiff reagent following enzymatic degradation of soluble glycogen and periodate oxidation of vicinal hydroxyl groups present in the insoluble form of glycogen (polyglucosan). Their presence in skin tissue enables LD diagnosis based on the minimally invasive skin biopsy [12,13]. Those in the brain are considered to trigger neurodegeneration and epileptic symptoms [14].

**Figure 1:**
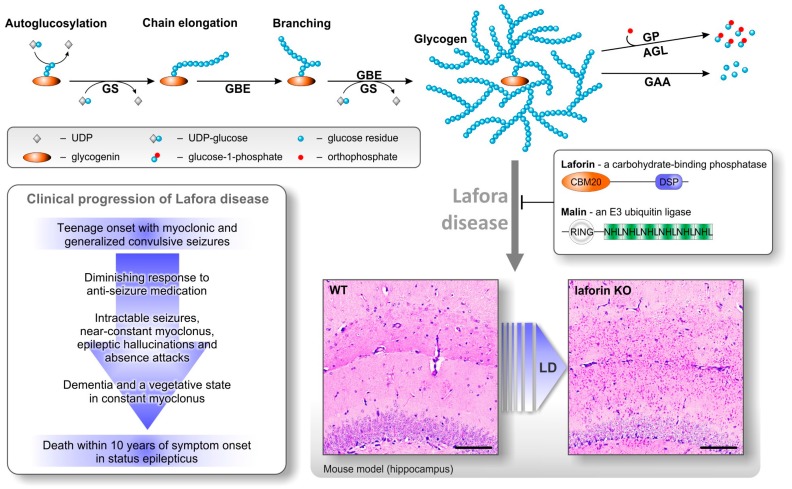
Lafora disease (LD) in the context of glycogen metabolism. Synthesis of glycogen molecules in the cytosol (top row) essentially includes autoglucosylation of glycogenin, elongation of chains by glycogen synthase (GS), and introduction of branching points by glycogen branching enzyme (GBE). The regularly branched and water-soluble glycogen molecules are degraded by glycogen phosphorylase (GP) and glycogen debranching enzyme (AGL, indirect debranching) in the cytosol or by lysosomal enzymes such as acid α-glucosidase (GAA). Functional laforin and malin essentially inhibit the formation of Lafora bodies and prevent LD as indicated by the inhibitory arrow. Mutations leading to non-functional laforin or malin (protein domains explained in the text) result in formation and accumulation of Lafora bodies as exemplarily shown in brain sections of the laforin-deficient mouse model (bottom right, scale bar equals 100 µm). In humans, LD manifests in an eventually fatal progression of clinical symptoms (bottom left).

Unfortunately to date there is no cure available for LD, with treatment being limited to alleviating the myoclonus epilepsy via antiepileptic drugs [15]. Understanding of the mechanisms underlying the pathogenesis of LD is paramount for the development of appropriate treatment strategies (Figure 1).

## 2. Lafora Bodies Put Lafora Disease in the Context of Polyglucan Metabolism

Consisting mostly of polyglucan chains, and to a minor extent proteins [16], the water-insoluble LBs are historically classified as polyglucosan bodies. Sensitivity of LBs to in vitro amylolysis implies the presence of α-(1,4) and α-(1,6) glucosidic linkages [17]. Both interglucose bonds are the essential linkages in water-soluble glycogen as well as in water-insoluble plant starches. The chemical similarity of the main constituent of LBs (polyglucosan) and soluble glycogen already indicates a close relation between glycogen metabolism and the formation of LBs (Figure 1). It is obvious that, despite similarities in the chemical composition of all these polyglucans, other features determine their (in)solubility in water.

Glycogen, serving as a cellular storage for reduced carbon and energy, is a regularly branched polymer of up to 55,000 glucosyl residues per molecule (β-particle). By a defined association of several β-particles even bigger assemblies (α-particles) can be formed, which have so far been described in liver [18] and heart [19] tissue. Glycogen is a disperse mixture of molecules with a wide range of molecule sizes [18,20,21]. Within glycogen molecules, chains have a distribution of lengths ranging from a degree of polymerisation of three to more than thirty-five [20,22], with an average length of approximately thirteen glucosyl residues [23].

De novo synthesis of glycogen molecules mainly involves autoglucosylation by glycogenin utilizing uridine 5’-diphosphoglucose (UDP-glucose) [24]. Recent results, however, suggest that glycogenin is not strictly necessary for glycogen synthesis [25]. Chains are elongated at the non-reducing end by glycogen synthase (GS) using UDP-glucose. Branching points are introduced by glycogen branching enzyme (GBE). Degradation is mediated by glycogen phosphorylase (GP) and glycogen debranching enzyme (AGL, see Figure 1) [24]. The enzymatic apparatus for glycogen turnover in mammals appears to be simple, especially when compared to the complexity of higher plant polyglucan (starch) metabolism, where several isoforms of synthesizing and degrading enzymes are expressed in a single cell [26]. However, in glycogen metabolism, additional enzymatic complexity is achieved, predominantly by regulatory features of the enzymes involved. For instance, while comparatively little is known about regulatory features of GBE, GS is strongly regulated by both covalent modifications and allosteric effects. Covalent modifications are essentially phosphorylations at multiple sites that partly occur in a hierarchical order. Increasing GS phosphorylation, as mediated by protein kinases such as glycogen synthase kinase 3 (GSK3), leads to an inactivation of GS. In turn, dephosphorylation by protein phosphatase 1 (PP1) activates GS [24]. Allosteric binding of glucose 6-phosphate (G6P) overrides the inhibition as imposed by GS phosphorylation and can restore full GS activity [27]. The complexity of GS regulation is increased by additional factors. First, main regulators of GS (GSK3 and PP1) are regulated themselves on a higher level by processes that include protein-(de)phosphorylation [28], binding to targeting subunits [29], and ubiquitination [30]. Secondly, concentrations of cellular constituents, such as UDP-glucose and/or of the allosteric activator G6P (and GS itself) may not be constant at all times and equal at all loci in the cell but underlie spatial and/or temporal variations. Interestingly, subcellular variations have been demonstrated for a variety of low molecular compounds such as O_2_, adenosine triphosphate (ATP), reduced nicotinamide adenine dinucleotide (NADH), glucose, Ca^2+^, and H^+^ [31,32,33]. Thus, during glycogen synthesis a certain degree of heterogeneity regarding the kinetic properties of individual GS molecules within the GS population of a single cell is likely to exist. This may contribute to the heterogeneity of structural properties observed in glycogen preparations, such as different chain lengths and molecule sizes.

In LD mouse models (laforin or malin knockout (KO), glycogen preparations have been shown to differ from wild-type (WT) by having, on average, longer glucan chains as well as by containing higher levels of covalently bound phosphate [20,34,35]. Important questions are (1) how the absence of laforin or malin inflicts these changes in glycogen structure, and (2) whether these changes contribute to glycogen insolubility, LBs accumulation, and LD.

## 3. Lafora Bodies—Cause or Effect of Lafora Disease?

The relatively slow progression of LD is consistent with a slow accumulation of LBs. However, the first question to be answered is whether LBs are actually pathological to, or merely symptomatic of LD. While there has been some disagreement in the field, the evidence is building that LBs are causative of LD. First, diminishing glycogen synthesis in malin KO mice by partial or full GS knockout revealed that decreasing glycogen and LB accumulation prevents the neurodegeneration and impaired autophagy seen in malin KO mice [14]. Furthermore, the knockout of PTG (protein targeting to glycogen, Ppp1r3c), a protein involved in the activation of GS, in laforin and malin-deficient mice prevented LB formation, seizure susceptibility, and neurodegeneration [36,37]. By contrast, an increase of total GS activity by overexpression of PTG in WT animals leads to accumulation of polyglucosan bodies and impaired autophagy [14], similar to what has been observed in mouse models of LD [14,38]. PTG overexpression in malin-deficient mice enhances both the glycogen accumulation and the observed autophagy phenotype [14]. Likewise, overexpression of constitutively active GS in WT mouse neurons results in accumulation of insoluble glycogen and neurodegeneration [39]. This strongly suggests that glycogen accumulation into LBs is a crucial process in the development of LD rather than a secondary effect. In turn it is possible that impaired autophagy is secondary to LB accumulation. As there is evidence that impaired autophagy can cause neurodegeneration [40,41,42], it appears to be possible that autophagy impairment is the link between LBs and the severe neurodegeneration of LD patients and mouse models. As the accumulation of LBs appears fundamental to LD, it is essential to understand how laforin-or malin-deficiency cause the accumulation of an insoluble form of glycogen (polyglucosan).

## 4. Laforin—A Carbohydrate-Binding Dual Specificity Phosphatase

Human laforin is encoded by *EPM2A*, a four-exon gene located on chromosome 6q24, resulting in a cytosolic protein of 331 amino acids [43]. Due to alternative splicing, additional isoforms may occur [44]. Within the laforin sequence, a putative dual specificity phosphatase domain (DSP) has been identified [1]. DSP domains are common in the large family of protein phosphatases capable of dephosphorylating phosphoproteins at the amino acid residues threonine/serine and tyrosine. Some DSP-containing phosphatases act also on various non-proteinaceous substrates [45]. In addition to the C-terminal DSP, laforin contains an N-terminal carbohydrate-binding module (CBM, family 20), which makes laforin to date the only protein in the animal kingdom to contain both a CBM and a DSP domain (Figure 1). The CBM promotes targeting of laforin to glycogen molecules, also locating the enzyme within the vicinity of glycogen metabolizing enzymes [46,47]. Given that LD is characterized by the accumulation of insoluble glycogen-derived LBs, it appears logical that CBM-mediated targeting of laforin to glycogen is crucial in preventing LB formation.

After identification of the laforin gene and its putative protein domains, the search was on for what key enzymes laforin dephosphorylates and how the lack of this leads to LD [1]. One proposed mechanism includes the GS regulator, glycogen synthase kinase 3 (GSK3) [48]. When overexpressing both laforin and GSK3β (isoform predominantly expressed in muscle) in Chinese hamster ovary (CHO) cells, two interesting results were obtained: Firstly, the two proteins interacted. Secondly, laforin was able to greatly reduce the amount of phosphate esters at residue Ser 9 of GSK3β [48]. This is expected to activate GSK3β and thereby to deactivate GS through increased phosphorylation [24].

**Figure 2:**
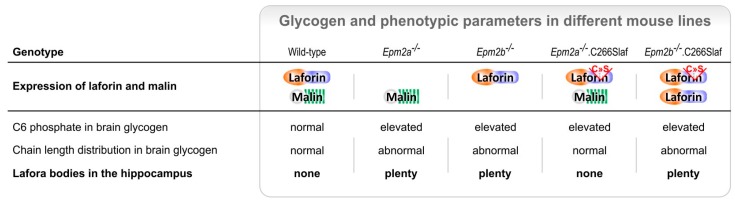
Prevention of LBs depends on the presence of malin but not on laforin’s phosphatase activity. The phenotypic features (C6 phosphate and chain length distribution, CLD) of brain glycogen as well as LB occurrence in the hippocampus are summarized for different LD-related mouse lines. Overexpression of phosphatase inactive laforin (C266S mutation in the DSP domain) leads to glycogen hyperphosphorylation but prevents both CLD abnormality and LB accumulation provided malin is functional. Hyperphosphorylation per se does not cause LB formation and accumulation.

Another interesting discovery was that laforin can function as a glucan phosphatase [49], removing the small amounts of phosphate groups attached to glycogen [34]. This finding, along with the hyperphosphorylation of glycogen from laforin KO mice [34], led to the hypothesis that elevated levels of phosphate esters cause the abnormal glycogen structure and trigger LB formation [35,50]. The phosphatase activity of laforin, however, appears not to be required to prevent the disease. This was concluded from the absence of the LD phenotype in mice lacking endogeneous laforin but overexpressing a mutated form of the protein (C265S in murine, C266S in human laforin) that lacks the phosphatase activity [38]. A more recent study clearly showed that muscle as well as brain glycogen from those mice is still hyperphosphorylated but the chain length distribution of glycogen is completely normalized in both tissues [51]. These results indicate that glycogen hyperphosphorylation does not cause the formation of LBs (Figure 2), making the search for a different cause paramount to the understanding of this disease.

The list of potential laforin interaction partners is still increasing [43]. Besides GSK3β [48], this list includes malin [52], three regulatory subunits of protein phosphatase 1 (PP1, the subunits are PTG [52], G_L_ [49], R6 [30]), GS [49], the α and β unit of adenosine monophosphate-activated protein kinase (AMPK) [53], the proteins tau [54], Hira-interacting protein 5 (HIRIP5) [55], and EPM2A-interacting protein 1 (EPM2AIP1) [56]. These proteins appear to be related to glycogen metabolism, but also to endoplasmic reticulum stress as well as protein clearance, iron homeostasis, and tumor suppression. While the significance of some of the reported interactions is still controversial, it is clear that the role of laforin is complex and most likely involves multiple functions.

The interaction of laforin with malin and their collaborative function is supported by a large number of studies [30,48,52,53,57,58]. In most of these studies, both proteins were fused to an epitope tag and overexpressed in cell culture, their interaction having been demonstrated by co-immunoprecipitation. The biological relevance of protein-protein interactions using these experimental settings can be questioned due to the unrealistically high protein concentrations and the difficulties to replicate biologically representative environments. However, the existence of LD patient mutations (e.g., D146N in malin) that cause a loss of malin-laforin interaction [58] strongly suggests an interaction of both proteins in vivo. Furthermore, a functional laforin-malin complex being essential to prevent LD is in line with the generally indistinguishable clinical outcomes when one of the two interacting partners is non-functional. Many details about the laforin-malin complex such as the in vivo stoichiometry of the binding partners remain elusive but might be mechanistically relevant. To a large extent, the limited knowledge is due to the absence of a suitable antibody against malin.

## 5. Malin—An E3 Ubiquitin Ligase

Human malin, encoded by *EPM2B* (also called *NHLRC1*), a single-exon gene located on chromosome 6p22.3, is a 395 amino acid protein that contains a RING and six NHL-repeat domains, the former strongly suggesting an E3 ubiquitin ligase activity [59,60]. As in the case of laforin-mediated protein dephosphorylation, putative target proteins were sought that are ubiquitinated by malin. It was empirically confirmed that malin indeed functions as E3 ubiquitin ligase, with the capability of self-ubiquitination under in vitro conditions, a feature shared with other E3 ligases [48].

There is evidence that the laforin-malin complex predominantly incorporates K63-linked polyubiquitin chains [61,62,63]. This type of modification of target proteins appears to promote their autophagic inclusion and degradation [64,65,66]. However, it was also shown that malin-dependent target degradation can be counteracted by inhibitors of the proteasome [30,58]. One publication also demonstrates malin-dependent incorporation of K48-linked polyubiquitin [67] which suggests proteasomal targeting. Thus, it is still unclear whether targets of the laforin-malin complex are subjected to the cytosolic proteasome or to autophagy (or both). However, it seems very likely that malin targets are subjected to degradation.

Malin has been shown to ubiquitinate laforin, thereby promoting its degradation [68]. Furthermore, malin appears to be capable of ubiquitinating both GS [58] and AGL [69], but also PTG [30], an important scaffold protein that targets several enzymes to glycogen. This is, for example, the case for PP1, which by dephosphorylation leads to activation of GS [29]. In addition, the laforin-malin complex has been reported to ubiquitinate AMPK, a sensor of cellular energy stores [61], as well as both pyruvate kinase 1 and 2 [70]. For most of the proposed targets of the laforin-malin complex, however, ubiquitination and its down-stream effects have not been conclusively demonstrated at physiological concentrations in vivo. Doubt has been raised whether PTG, AGL and GS are substrates for malin ubiquitination, with reports of no evidence of altered levels of these proteins observed in either laforin or malin KO mice [71]. Some uncertainties are, however, inherent to these studies. Firstly, the total amount of any protein of interest in a tissue lysate, as analyzed by Western blotting, may not reflect changes of a small proportion of that protein, for example occurring on a subcellular level. Secondly, biological variation may require a higher number of biological replicates for a slight difference between genotypes to reach statistical significance. For instance, it has been shown that PTG levels in the muscle, while not reaching significance, were slightly increased (approximately by 20%) in malin KO mice compared to WT mice in the insoluble fraction of tissue lysates, as obtained in the so-called high speed pellet [71]. This may indicate an association of PTG with insoluble LBs, implying the possibility of PTG being indeed a target of the laforin-malin complex in vivo.

In summary, the function of polyubiquitination by the laforin-malin complex thus seems to link autophagy-related processes to glycogen metabolism. It therefore remains to be discussed how both processes could lead to LB accumulation.

## 6. Factors That May Cause Glycogen Insolubility

A mutated laforin that possesses a catalytically inactive phosphatase site is able to prevent LD in mice deficient of endogenous laforin [38]. Moreover, hyperphosphorylation of glycogen is not strictly correlated with LB accumulation [51]. These results demonstrate that the increased glycogen phosphate levels per se cannot be the underlying cause of LD. The exact role of the glycogen phosphate remains to be elucidated.

**Figure 3:**
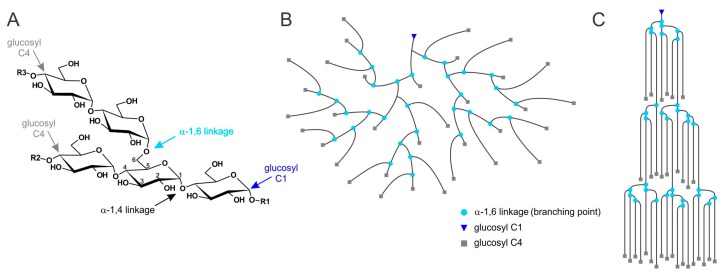
Branching structure of amylopectin and glycogen. (**A**) Chemical basis of a branched α-glucan. R1, glucan chain at the reducing end; R2 and R3, chains at the nonreducing ends; (**B**,**C**) Segments of glycogen and amylopectin, respectively. In glycogen, branching points are essentially distributed evenly; in amylopectin, they are clustered. In areas with a low abundance of branching points, double-helices form. Modified version from [20].

**Figure 4:**
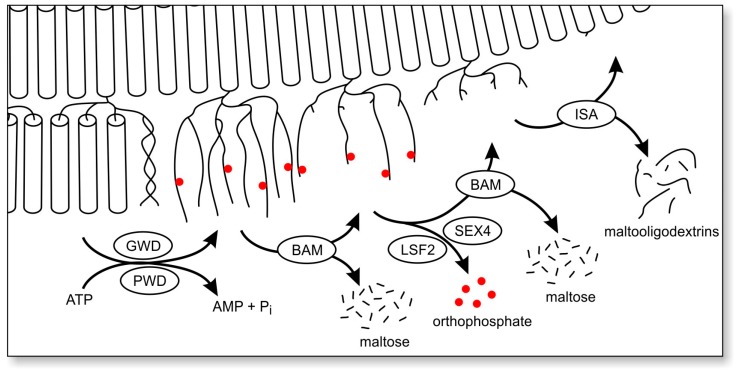
Role of phosphate esters during degradation of plant amylopectin. Degradation of starch granules in higher plants is facilitated by cycles of phosphorylation and dephosphorylation. Tightly packed, semi-crystalline exterior glucan chains of amylopectin undergo phase transition (specified in the text) through incorporation of phosphate esters by glucan water dikinase (GWD) and phosphoglucan water dikinase (PWD), both utilizing ATP. Exo-acting β-amylases (BAM) remove maltose units from the non-reducing ends, their action being obstructed by phosphate esters and branching points. The glucan phosphatases SEX4 (starch-excess 4) and LSF2 (Like-SEX4 2) remove phosphate esters and enable further BAM-mediated degradation. Isoamylase (ISA) is required for direct debranching in the amorphous layer of amylopectin before GWD-mediated phosphorylation initiates degradation of the subsequent semi-crystalline layer. Figure modified from [47,78].

It may, however, be informative to summarize the current knowledge of (de)phosphorylation of another polyglucan-storing biological system, i.e., plant starch metabolism. Typically, plants form and mobilize water-insoluble starch granules as a functional equivalent to glycogen in animals [72]. With the exception of some mutants, plant starch contains two types of polyglucans, amylopectin and amylose. Amylopectin is the dominant polyglucan that defines most of the properties of the entire starch particle. Furthermore, amylopectin possesses a relatively high content of branching points, a higher average molecular weight, and is, therefore, more similar to glycogen as compared to amylose (Figure 3) [73,74]. Phosphate esters are introduced into amylopectin by distinct dikinases, both during synthesis and degradation [75,76]. It is widely accepted that the covalent modification by phosphorylation mediates a reversible (and local) transition from a highly ordered but less hydrated form of the polyglucan into a less ordered but more hydrated form of starch [47,77,78]. During degradation one established role of phosphate esters is, due to the phosphate’s high charge and hydrophilia, to separate double-helices from each other, rendering them fully hydrated and, thus, facilitating access of starch degrading enzymes (Figure 4) [77,78]. As both glycogen and amylopectin have a common chemical basis, both being composed of branched α-glucan chains, local physicochemical effects of branching points or of similar covalent modifications (such as phosphorylation), are expected to have, in principle, similar consequences. Regarding what is known from starch metabolism, phosphorylation of glycogen should actually counteract glycogen insolubility. This implies that in the pathological LD state hyperphosphorylation would be beneficial rather than harmful.

Another factor that has been discussed as causative of LB accumulation is simply the cellular glycogen concentration. This hypothesis is supported by the fact that accumulation of insoluble glycogen (polyglucosan) is observed as a result of increased GS activity by overexpression of PTG or of GS itself [14,39]. While the cellular concentration of brain glycogen in these PTG or GS overexpressing mice is significantly higher than in the controls, these values are still 30–100 times lower than what is observed in healthy liver tissue [79]. This, together with the fact that in the brain LBs are mostly formed in neurons which have much lower glycogen contents than for instance astrocytes [80], makes it unlikely that glycogen becomes insoluble simply due to an increased concentration.

By contrast, the abnormal chain length distribution of glycogen strictly correlates with the occurrence of LBs in laforin- and malin-deficient mice [51]. The relevance of the chain length and branching pattern for the hydrosolubility of glucan polymers is obvious when comparing the solubility of the different polyglucans, starch, glycogen, and phytoglycogen. The latter is found in some plants or plant tissues with an altered expression of a few starch-related enzymes. These alterations are mostly biotechnologically induced (mutants) but can also occur naturally [81,82]. Non-pathological glycogen, amylopectin, and phytoglycogen all consist of chains of glucosyl residues connected via α-(1,4) linkages, which contain branch points via α-(1,6) linkages [83,84]. Glycogen and phytoglycogen are water-soluble while amylopectin is not. The average chain length of amylopectin varies depending on the plant species and tissue but is generally much higher than that of glycogen and phytoglycogen [85]. However, while in the soluble polyglucans the branching points are equally distributed throughout the molecule, in amylopectin they form clusters (Figure 3) [73,74]. The clustered arrangement gives rise to areas with low numbers of branching points. In these areas, chains have been shown to occur as double-helices including all but a few glucosyl units that are closest to the branch point and, therefore, are excluded from the double-helix due to steric constraints. Tight packing of the double-helices induces crystallization, hence water-insolubility of the highly dense starch particles [73,85,86]. Using purified malto-oligosaccharides as polyglucan models, the minimum length for glucan chain crystallization is reported to be 10 glucose units. In the presence of longer chains, however, malto-oligosaccharides of at least 6 units can co-crystallise [87].

Thus, it is obvious that frequent and regularly distributed branching points lead to shorter chains and less double-helix formation. Indeed, a strong correlation of chain length distribution and polyglucan crystallinity has been demonstrated using a range of mutant starches. In these studies, a high abundance of short glucan chains was consistently found to interfere with polyglucan crystallinity [88,89]. Furthermore, phytoglycogen-producing mutant plants accumulate insoluble polyglucans when additionally overexpressing a chain-elongating starch synthase, leading to (1) a higher abundance of longer glucan chains, and (2) to a substantial increase in polyglucan crystallinity as induced by double-helix formation [82].

A clear difference between water-soluble normal glycogen and the partially insoluble LD glycogen is the enrichment of longer α-glucan chains in the latter, as seen by its abnormal chain length distribution [20]. Similar to LD-related glycogen insolubility, a deficiency of GBE also leads to a dramatic accumulation of insoluble glycogen in the mouse model of GBE deficiency [90]. In turn, overexpression of GS or PTG, both leading to increased GS activity, results in polyglucosan body formation in mouse muscle and brain [39,91]. Thus, evidence is compelling that the abnormal chain length distribution of glycogen (i.e., the higher abundance of longer glucan chains) is the general reason for glycogen insolubility in LD.

**Figure 5:**
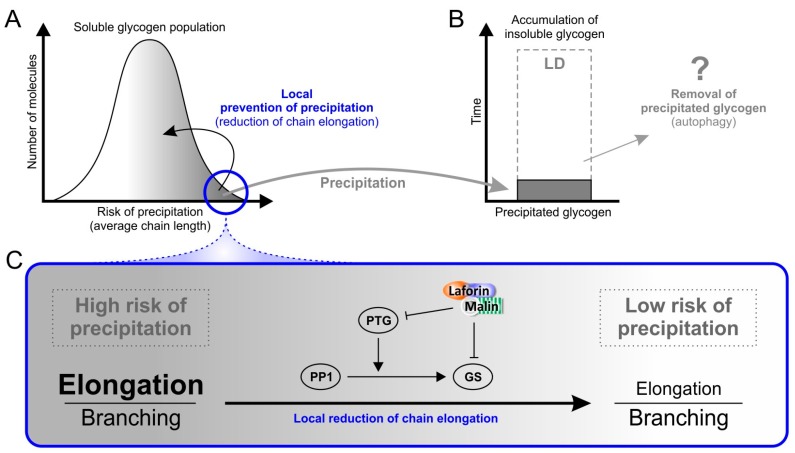
Mechanistic model for Lafora body formation and accumulation. (**A**) Glycogen as a disperse mixture of molecules that possess different contents of long glucan chains and according risks of precipitation. The minor proportion of molecules (blue circle) actually subject to precipitation (grey arrow) may be modified by local reduction of chain elongation that is achieved by a well-supported mechanism explained in (**C**). This modification increases the relative branching frequency and decreases the risk to precipitate; (**B**) If glycogen precipitation is not (entirely) avoided, precipitated glycogen is accumulating over time (*y*-axis) as seen in Lafora disease, unless cells are able to mobilize and remove precipitated glycogen (e.g., hypothetically (question mark) through autophagy) at a rate that is higher than that of precipitation; (**C**) The mechanism by which a functional laforin-malin complex can reduce GS activity as supported by experimental data obtained in cell-culture [30,58]. Negative effects are indicated by inhibitory arrows while normal arrows indicate a positive effect. By targeting GS and PTG to degradation, the laforin-malin complex reduces GS activity and relatively increases branching frequency. It is proposed that this process may not significantly affect the entire GS population but is occurring locally, i.e., predominantly at a minor proportion of glycogen molecules with a high risk of precipitation (blue circle in (**A**)).

It should be noted that the chain length distribution has to date been determined as an average of all glycogen molecules present in the respective tissue sample. At a subcellular/submolecular level, minor differences in the activities of GS and GBE may be part of normal glycogen metabolism. Such localized differences could lead to variations of chain length distributions in individual molecules and/or even in some regions of a given glycogen molecule, but remain undetected by conventional chain length distribution analyses. There is, however, evidence that even in wild-type mice some glycogen molecules possess longer chains than others, i.e., that not all molecules within one cell have an identical chain length distribution [20,92]. It is therefore possible that in the disperse mixture of glycogen molecules within a cell, not all molecules are affected to the same extent by the chain length abnormality seen in LD. Taking into account that the chain length greatly determines polyglucan solubility (see above), it implies that not all glycogen molecules may have the same risk (i.e., probability) to precipitate. Precipitation of glycogen likely affects a small proportion of glycogen molecules that have the most severe chain length abnormality. By contrast, less affected glycogen molecules (which probably represent the vast majority) can still be metabolized normally (Figure 5A). The hypothesized scenario is fully in line with the fact that insoluble polyglucosan, seen in LD, accumulates slowly.

## 7. Why Does Insoluble Glycogen Accumulate in Lafora Disease?

With a loss-of-function mutation in either laforin or malin causing essentially the same pathological phenotype, and the strong evidence for in vivo laforin-malin interaction [48,53,68,93], it seems likely that the primary cause of LD is the lack of a functional laforin-malin complex. One function of this complex must therefore be associated with the avoidance of LB accumulation. From a mechanistic point of view, LBs are accumulating when the rate of production exceeds the rate of removal of the insoluble glycogen that aggregates in LBs. To accumulate bodies over time, the rate of removal can be substantial or infinitely small, but, in any case, must be smaller than the rate of production. In principle, deficiency in the laforin-malin complex may lead to an increased formation, or to an impaired removal of LBs, or may even affect both processes (Figure 5A,B). However, there is yet no evidence for a mechanism to remove LBs in vivo.

While plants possess the capacity to mobilize water-insoluble polyglucans (starch) under a wide range of external conditions, this is certainly not the case in animals, which generally have evolved to use water-soluble polyglucans (glycogen). Insolubility and aggregation of glycogen can have severe consequences in mammalian cells which becomes obvious in the neurons of LD patients. Thus, it is likely that hypothetical mechanisms to dispose of insoluble glucan material work at a low rate and may practically render precipitated glycogen metabolically inert.

Avoiding precipitation altogether would be biologically important. One possibility is that the laforin-malin complex is able to decrease GS activity selectively on molecules that are likely to precipitate, for example molecules that are still soluble but are, on average or in particular molecule regions, composed of longer glucan chains (Figure 5A). Indeed, it has been shown that laforin preferentially binds solubilized potato starch as compared to glycogen in vitro and polyglucosan bodies relative to glycogen in vivo [94], implying a preference of laforin for substrates composed of longer glucan chains and possessing less branching points. One role of laforin in the complex with malin would be to target malin via its CBM to a subset of glycogen molecules that possess abnormally long chains and are at a higher risk to precipitate. By polyubiquitination, malin promotes targeting of GS and PTG to degradation, whereby it could locally decrease chain elongation. Continued glycogen synthesis with locally reduced GS activity may lead to an increased frequency of branching points promoting water-solubility. Consequently, the risk of precipitation is diminished for distinct glycogen molecules that were otherwise prone to precipitate (Figure 5A,C). This model of locally focussed action of the laforin-malin complex consolidates the apparently conflicting results that (1) malin ubiquitinates PTG [30,58] and GS [58] leading to their degradation, and (2) total GS and PTG levels are not significantly altered in LD mice [71].

However, a mechanism for the removal of abnormal and insoluble glycogen cannot be ruled out and may also involve a functional laforin-malin complex. One possible mechanism of removal could proceed via autophagy which has been shown to be impaired in some LD mouse models but not in others [22,38,51]. There is increasing evidence that accumulation of insoluble glycogen underlies the inconsistently detected general autophagy impairment [14,95]. In cell culture studies, a laforin-malin complex has been associated with two autophagy-related proteins, designated LC3 and p62. The first is located in the autophagosome membrane, the second is an adapter protein that interacts with the laforin-malin complex and binds ubiquitinated proteins that are targeted to autophagy [57]. It has been shown that a general defect of autophagy cannot be strictly correlated with LB formation, and hence is not the primary cause of LD [22,51,96]. Nevertheless, it is possible to hypothesize that autophagy is involved in LB removal (Figure 5B). This process may be dependent on a functional laforin-malin complex and precisely act to target and dispose of abnormal and precipitated glycogen (Figure 5). Its impairment in LD mouse models may not consistently and detectably affect markers of general autophagy.

Thus, the function of the laforin-malin complex may be implicated in (1) a locally focussed alleviation of glycogen precipitation risks, (2) a selective removal of abnormally formed and precipitated glycogen, or (3) in both. All of these hypotheses are unified in the assumption of an underlying intrinsic property of glycogen metabolism to inadvertently produce disadvantageous structural variations of glycogen molecules including some that are at risk to precipitate. The hypotheses differ in how they assume the cell copes with the risk of glycogen precipitation. While according to the first hypothesis, glycogen precipitation is avoided as much as possible, the second hypothesis implies a removal of abnormal and precipitated glycogen by autophagy. It is not inconceivable that mammalian cells employ both of these strategies at the same time (hypothesis 3, Figure 5).

## Abbreviations

AGL	Glycogen debranching enzyme
AMPK	AMP-activated protein kinase
CBM	Carbohydrate-binding module
CLD	Chain length distribution
DSP	Dual specificity phosphatase
G6P	Glucose-6-phosphate
GBE	Glycogen branching enzyme
GP	Glycogen phosphorylase
GS	Glycogen synthase
GSK3	Glycogen synthase kinase 3
KO	Knockout
LB	Lafora body
LD	Lafora disease
PP1	Protein phosphatase 1
PTG	Protein targeting to glycogen – regulatory subunit of PP1
WT	Wild-type
